# RNAase III-Type Enzyme Dicer Regulates Mitochondrial Fatty Acid Oxidative Metabolism in Cardiac Mesenchymal Stem Cells

**DOI:** 10.3390/ijms20225554

**Published:** 2019-11-07

**Authors:** Xuan Su, Yue Jin, Yan Shen, Il-man Kim, Neal L. Weintraub, Yaoliang Tang

**Affiliations:** 1 Vascular Biology Center, Medical College of Georgia, Augusta University, Augusta, GA 30912, USA; XSU@augusta.edu (X.S.); YUJIN@augusta.edu (Y.J.); YASHEN@augusta.edu (Y.S.); NWEINTRAUB@augusta.edu (N.L.W.); 2 Department of Anatomy, Cell Biology & Physiology, School of Medicine, Indiana University, Indianapolis, IN 46202, USA; ilkim@iu.edu

**Keywords:** dicer, mitochondrial oxidative metabolism, cardiac mesenchymal stem cells (C-MSC), aerobic glycolysis, β-oxidation, Warburg effect

## Abstract

Cardiac mesenchymal stem cells (C-MSC) play a key role in maintaining normal cardiac function under physiological and pathological conditions. Glycolysis and mitochondrial oxidative phosphorylation predominately account for energy production in C-MSC. Dicer, a ribonuclease III endoribonuclease, plays a critical role in the control of microRNA maturation in C-MSC, but its role in regulating C-MSC energy metabolism is largely unknown. In this study, we found that Dicer knockout led to concurrent increase in both cell proliferation and apoptosis in C-MSC compared to Dicer floxed C-MSC. We analyzed mitochondrial oxidative phosphorylation by quantifying cellular oxygen consumption rate (OCR), and glycolysis by quantifying the extracellular acidification rate (ECAR), in C-MSC with/without Dicer gene deletion. Dicer gene deletion significantly reduced mitochondrial oxidative phosphorylation while increasing glycolysis in C-MSC. Additionally, Dicer gene deletion selectively reduced the expression of β-oxidation genes without affecting the expression of genes involved in the tricarboxylic acid (TCA) cycle or electron transport chain (ETC). Finally, Dicer gene deletion reduced the copy number of mitochondrially encoded 1,4-Dihydronicotinamide adenine dinucleotide (NADH): ubiquinone oxidoreductase core subunit 6 (MT-ND6), a mitochondrial-encoded gene, in C-MSC. In conclusion, Dicer gene deletion induced a metabolic shift from oxidative metabolism to aerobic glycolysis in C-MSC, suggesting that Dicer functions as a metabolic switch in C-MSC, which in turn may regulate proliferation and environmental adaptation.

## 1. Introduction

Cardiovascular diseases are the leading cause of morbidity and mortality worldwide [1]. In patients with heart failure, disorders of substrate utilization and intermediate metabolism, energy deficiency, and oxidative stress underlie the basis of systolic dysfunction and disease progression [2]. Mitochondria control many biological processes, most importantly, aerobic metabolism to generate ATP, a process known as oxidative phosphorylation (OXPHOS). In the normal adult heart, about 70% of ATP production is derived from fatty acid oxidation [3]. To sustain sufficient energy production required to support the continuous mechanical workload, however, the heart utilizes diverse energy substrates, including glucose and amino acids [4]. Fatty acid oxidation is a complex biological process involving including mitochondrial β-oxidation, tricarboxylic acid (TCA) cycle activity, and the electron transport chain (ETC) [5]. Defects in the OXPHOS system can cause a variety of cardiovascular diseases, such as ischemic heart failure, and diabetic cardiomyopathies, in which cardiac mitochondrial dysfunction and impaired energy production have been observed, potentially contributing to a decrease in contractile function and cardiac efficiency [5,6,7].

Tissue-resident mesenchymal stem cells play an essential role in maintaining normal tissue function under physiological and pathological conditions. Cardiac mesenchymal stem cells (C-MSC) reside in the heart and express the early cardiac-specific transcription factor GATA4 and mesenchymal stem cell markers, including CD105, CD140, and Sca-1 [8,9]. C-MSC transplantation improved heart repair in ischemic myocardium via secretion of exosomes [8,10] and bioactive proteins such as VEGF and SDF-1α [11]. However, little is known about the metabolism of C-MSC contained in adult hearts.

MicroRNAs (miRNAs) are small, noncoding RNAs (containing about 22 nucleotides) that function in regulating gene expression, mostly at the posttranscriptional level [12]. Additionally, miRNAs have been implicated in the regulation of energy metabolism in previous studies [13,14,15]. The biogenesis of miRNAs is under tight regulatory control; in particular, an RNase III endoribonuclease, Dicer, plays an indispensable role in cleaving pre-miRNAs into mature miRNAs that are loaded onto the RNA-induced silencing complex (RISC) in the cytoplasm to exert their biological function [16]. Because Dicer directly affects the final process of miRNA maturation, it is essential for maintaining normal heart development [17], stem cell proliferation and differentiation [18,19], and normal cardiac function [20]. Dicer expression is essential for postnatal cardiac development and function [21]. Dicer expression was reported to be reduced in end-stage human dilated cardiomyopathy and heart failure; conversely, Dicer expression was reported to be increased in the hearts of patients with improved cardiac function after implantation of left ventricle assist devices [20]. Alterations in cardiac metabolism are considered as a critical feature in the pathophysiology of cardiac dysfunction, in which cardiac metabolism switches from fatty acid oxidation to anaerobic glycolysis [5]. However, little is known about the role of Dicer in regulating C-MSC metabolism. We hypothesized that down-regulation of Dicer expression in C-MSC modulates cellular metabolism to accommodate diminished oxygen and substrate supply associated with heart failure. In this study, we assessed the role of Dicer in the metabolism of adult mouse and human C-MSC. We found that Dicer deletion significantly reduces mitochondrial respiration and influences fatty acid β-oxidation by decreasing the expression of related enzymes in C-MSC.

## 2. Results

### 2.1. Characterization of C-MSC

**Figure 1:**
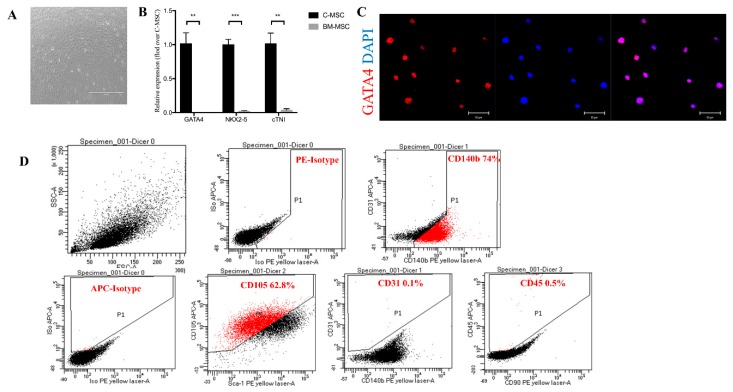
Characterization of cardiac mesenchymal stem cells (C-MSC). (**A**) Cultured C-MSC, scale bar = 1000 μm. (**B**) Comparison of mRNA expression of GATA4, NK2 homeobox 5 (Nkx2.5), and cardiac troponin-I (cTnI), three cardiac-specific genes, between C-MSC and bone marrow-derived MSC (BM-MSC). The amount of mRNA was normalized using β-actin (*n* = 3, ** *p* < 0.01, *** *p* < 0.001). (**C**) Immunofluorescent staining of C-MSC for the cardiac transcription factor GATA4 (red), scale bar = 50 μm. (**D**) Flow cytometric analyses of C-MSC for expression of the cell surface markers CD140b, CD105, CD31, and CD45.

C-MSC were grown from enzymatically digested adult mouse hearts and achieved approximately 80% confluence after 7–10 days in culture (Figure 1A). C-MSC express a significantly higher level of cardiac-specific genes, such as *GATA4*, *NK2* homeobox 5 (*Nkx2.5*), and cardiac troponin-I (cTnI) in comparison with bone marrow-derived MSC (BM-MSC) by qRT-PCR (Figure 1B). Immunofluorescent staining showed that C-MSC express GATA4, an early cardiac transcription factor (Figure 1C) [22], indicative of their cardiac origin. Surface marker expression was profiled by flow cytometry. Over 60% of cells were positive for CD105, and 74% cells were positive for CD140b, whereas only 0.1% were positive for CD31, and 0.5% for CD45 (Figure 1D). These data indicate that C-MSC represent a subpopulation of cardiac-derived mesenchymal stem cells [23].

### 2.2. Adenovirus-Mediated Cre Recombinase Enzyme (CRE) Deletion of Dicer in C-MSC

**Figure 2:**
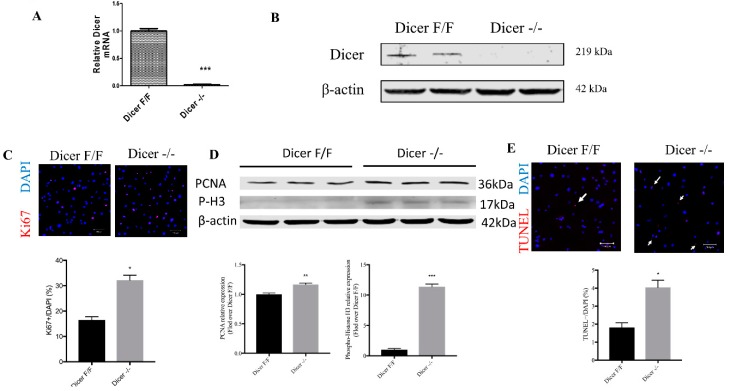
Adenovirus-mediated CRE recombinase expression resulted in a loss of Dicer expression in C-MSC. (**A**) Relative expression of Dicer mRNA. The amount of mRNA was normalized using β-actin. Results are shown as mean with standard error of the mean (SEM) (*n* = 3). (**B**) Western blotting of Dicer protein in adenovirus GFP (Ad-GFP) treated Dicer ^F/F^ C-MSC (Dicer ^F/F^), and adenovirus with CRE recombinase (Ad-CRE) treated Dicer ^F/F^ C-MSC (Dicer^−/−^) using β-actin as a loading control. (**C**) Comparison of the percentage of Ki67-positive cells between Dicer ^F/F^ and Dicer ^−/−^ C-MSC (scale bar= 100 µm) (* *p* < 0.05, *n* = 6). (**D**) Western blotting of PCNA and Phospho-H3 expression in Dicer ^F/F^ C-MSC (Dicer ^F/F^), and Dicer ^−/−^ C-MSC (Dicer ^−/−^) using β-actin as a loading control (** *p* < 0.01; *** *p* < 0.001, *n* = 3). (**E**) Comparison of the percentage of TUNEL-positive cells between Dicer ^F/F^ and Dicer ^−/−^ C-MSC (scale bar= 100 um) (*, *p* < 0.05, *n* = 20).

To delete Dicer in C-MSC, floxed Dicer C-MSC were infected with adenovirus expressing CRE recombinase (Dicer ^−/−^C-MSC); a GFP-expression adenovirus was employed as a control (Dicer^F/F^C-MSC). We determined whether infection of C-MSC with CRE recombinase resulted in a stable loss of Dicer mRNA and protein. As shown in Figure 2, infection with CRE recombinase resulted in the ablation of Dicer at both mRNA (Figure 2A) and protein (Figure 2B) levels. Cells infected with GFP or Cre recombinase were subsequently passaged for use in experiments. Immunofluorescent staining for Ki67 showed a twofold increase in Ki67 positive cells in the Dicer ^−/−^ C-MSC compared with Dicer ^F/F^ C-MSC (Figure 2C). Furthermore, we measured proliferating cell nuclear antigen (PCNA) and Phospho-H3 as mitotic markers by western blotting, as shown in Figure 2D, compared to Dicer floxed cells, and found that the knockout of Dicer in C-MSC increased the levels of both PCNA and P-H3 expression, indicating that the Dicer knockout increased cell proliferation. Next, we used terminal deoxynucleotidyl transferase dUTP nick end labeling (TUNEL) to evaluate cell apoptosis, observing by immunostaining approximately two fold higher TUNEL-positive Dicer ^−/−^ C-MSC compared with Dicer^F/F^ C-MSC (Figure 2E), indicating that Dicer ablation in C-MSC led to increased cell apoptosis.

### 2.3. Dicer Deletion Impairs Mitochondrial Respiration in C-MSC

**Figure 3:**
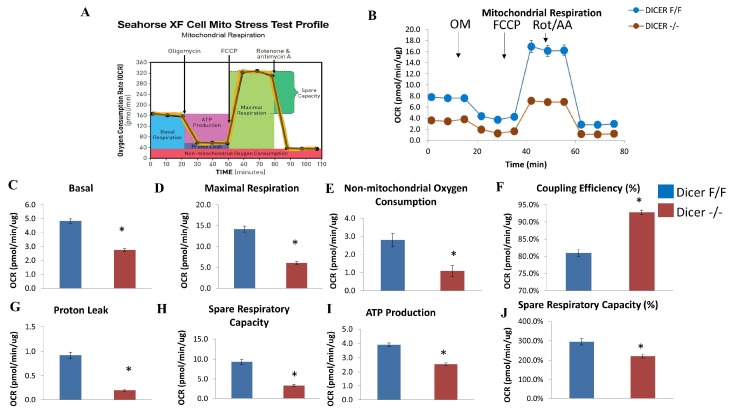
Assessment of oxygen consumption rate (OCR) in Dicer ^F/F^ and Dicer ^−/−^ C-MSC. (**A**) Schematic representation of the protocol employed in data collection and calculations of mitochondrial respiration. (**B**) Normalized OCR data. (**C**–**J**) Measurements of mitochondrial respiration calculated from the OCR tracing in (**B**). Results are normalized to total cellular protein and shown as mean ± SEM (*n* = 8), * *p* < 0.05. OM: Oligomycin; FCCP: carbonylcyanide 4-(trifluoromethoxy)-phenylhydrazone; Rot/AA: Rotenone & antimycin A.

To investigate whether the mitochondrial respiratory function in C-MSC is affected in response to Dicer gene deletion, we quantified cellular oxygen consumption rate (OCR), an index of oxidative phosphorylation. The injection of 1.25 µM oligomycin, 1 μM carbonylcyanide 4-(trifluoromethoxy)-phenylhydrazone (FCCP), and 0.5 µM rotenone/antimycin A allowed us to evaluate the sources and contribution of both mitochondrial and non-mitochondrial oxygen consumption. Figure 3A shows the protocol and calculations that were made with the injection of each compound. 

As shown in Figure 3B, Dicer ^−/−^ C-MSC exhibited a lower OCR compared with Dicer ^F/F^ C-MSC. Further calculation of the different components of oxygen consumption is shown in Figure 3C–J. Basal respiration, maximal respiration, non-mitochondrial respiration, proton leak, ATP production, and spare respiratory capacity were all significantly reduced in Dicer ^−/−^ C-MSC in comparison with Dicer ^F/F^ C-MSC. However, the coupling efficiency was higher in Dicer ^−/−^ C-MSC in comparison with Dicer ^F/F^ C-MSC. Taken together, these findings suggest that Dicer gene deletion results in decreased mitochondrial oxidative phosphorylation. 

### 2.4. Dicer Gene Deletion Increases Glycolysis in C-MSC

**Figure 4:**
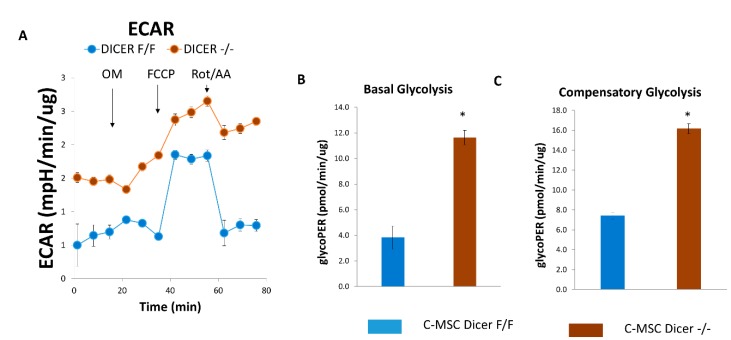
Assessment of extracellular acidification rate (ECAR) in Dicer ^F/F^ and Dicer ^−/−^ C-MSC. (**A**) Normalized ECAR data. (**B**,**C**) Measurements of basal glycolysis and compensatory glycolysis from the ECAR tracing in (**A**). Results are normalized to total cellular protein and shown as mean with SEM (*n* = 8), * *p* < 0.05.

Next, we quantified the extracellular acidification rate (ECAR), an index of cellular glycolytic capacity, in C-MSC using an established protocol [24]. Figure 4A–C shows that the ablation of Dicer in C-MSC increased basal and compensatory glycolysis compared to Dicer floxed C-MSC, suggesting that Dicer gene deletion led to increased glycolytic capacity in C-MSC. 

### 2.5. Dicer Deletion Decreases Mitochondrial Fatty Acid β-Oxidation in C-MSC

**Figure 5:**
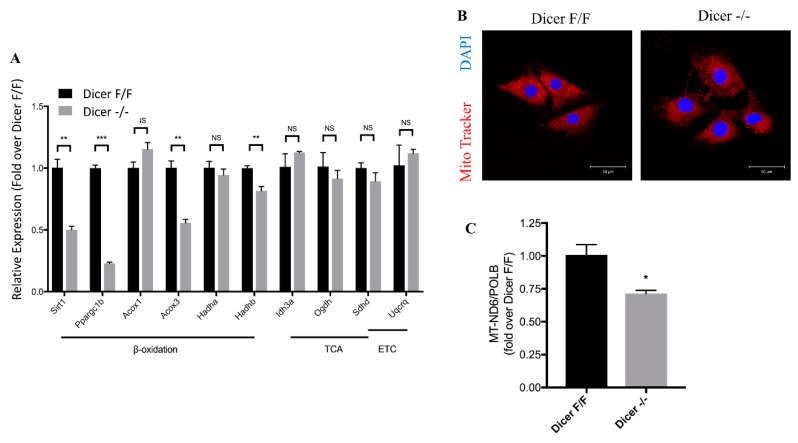
(**A**) Comparison of relative mRNA levels of genes related to mitochondrial fatty acid oxidation in mouse Dicer ^F/F^ and Dicer ^−/−^ C-MSC. The amount of mRNA was normalized using β-actin. TCA, tricarboxylic acid cycle; ETC, electron transport chain. Results are shown as mean ± SEM (*n* = 3), NS *p* > 0.05, * *p* < 0.05, ** *p* < 0.01, *** *p* < 0.001. (**B**) Representative MitoTracker Red CMXRos and DAPI staining of Dicer ^F/F^ and Dicer ^−/−^ C-MSC; confocal microscopy showed no remarkable change of mitochondrial mass detected by MitoTracker Red staining between Dicer ^F/F^ and Dicer ^−/−^ C-MSC (Scale bar= 50 µm). (**C**) Comparison of the relative amount of mitochondrially encoded 1,4-Dihydronicotinamide adenine dinucleotide (NADH): ubiquinone oxidoreductase core subunit 6 (MT-ND6) mitochondrial DNA (mtDNA) between Dicer ^F/F^ and Dicer ^−/−^ C-MSC. The amount of mtDNA was normalized to nuclear-encoded control gene DNA polymerase beta (PolB). (*n* = 4), * *p* < 0.05.

To further investigate the effect of Dicer deletion on fatty acid oxidative metabolism, we examined the expression of key fatty acid oxidation genes (Figure 5). Dicer ^−/−^ C-MSC exhibited decreased expression of genes related to fatty acid oxidation, such as sirtuin 1 (Sirt1), peroxisome proliferator-activated receptor gamma coactivator 1 beta (Ppargc1b), acetyl coenzyme A (acyl-CoA) oxidase 3 (Acox3), and hydroxyacyl-CoA dehydrogenase trifunctional multienzyme complex subunit beta (Hadhb), compared to Dicer ^F/F^ C-MSC, although no differences in expression were detected for acyl-CoA oxidase 1 (Acox1) and hydroxyacyl-CoA dehydrogenase trifunctional multienzyme complex subunit alpha (Hadha). Expression of genes related to the TCA cycle and ETC (e.g., isocitrate dehydrogenase (nicotinamide adenine dinucleotide (NAD)(+)) 3 Alpha (Idh3a), oxoglutarate dehydrogenase (Ogdh), succinate dehydrogenase complex subunit (DSdhd), and ubiquinol-cytochrome C reductase complex III subunit VII (Uqcrq)) was similar in Dicer ^F/F^ C-MSC and Dicer ^−/−^ C-MSC. These data suggest that Dicer plays an essential role in mitochondrial fatty acid oxidative metabolism by regulating genes that promote β-oxidation, but not oxidative phosphorylation, in C-MSC. Down-regulation of fatty acid oxidation genes following Dicer ablation suggests that Dicer may function as a metabolic switch in C-MSC. To determine whether the down-regulation of the fatty acid oxidation genes in response to Dicer ablation is conserved between mouse and human-derived C-MSC, we performed expression studies in Dicer small interfering RNA (siRNA)-treated human C-MSC and control (scramble siRNA-treated human C-MSC) by qRT-PCR, as shown in Figure S1, and Dicer expression was found to be down-regulated by approximately 75% by siRNA transfection in human C-MSC. Both Sirt1 and Ppargc1b were significantly down-regulated, although we detected no changes in expression of Acox3 or Hadhb. These findings suggest that Dicer regulation of fatty acid oxidation genes is at least partially conserved in C-MSC. We further investigated the effect of Dicer knockout on the mitochondrial structure using mitotracker red and DAPI staining. Both Dicer floxed and knockout C-MSC exhibited a normally well-defined mitochondrial shape, and no remarkable change of mitochondrial mass detected by MitoTracker Red staining between Dicer ^F/F^ and Dicer ^−/−^ C-MSC (Figure 5B). To quantify the number of mitochondria between Dicer ^F/F^ and Dicer ^−/−^ C-MSC, we extracted genomic DNA from C-MSC and measured relative mitochondrial and nuclear genome copy numbers by qPCR, as shown in Figure 5C. Compared to Dicer ^F/F^ C-MSC, Dicer ^−/−^ C-MSC exhibited approximately 28% reduced mitochondrial DNA content.

## 3. Discussion

In this study, we compared cellular metabolism and energetics in adult C-MSC following deletion of Dicer, a key enzyme involved in miRNA processing. Dicer gene deletion converted adult C-MSC from an oxidative state to a glycolytic state and selectively downregulated the expression of critical fatty acid oxidation genes, suggesting that Dicer functions as a metabolic switch in C-MSC. 

In our study, we demonstrated that knockout of Dicer in C-MSC increased the number of proliferating cells and protein levels of PCNA and p-H3, the markers of cell mitosis, in C-MSC compared to Dicer floxed cells, suggesting that Dicer knockout increased cell proliferation. Meanwhile, knockout of Dicer increased apoptosis occurred, as shown by TUNEL analysis. As we have seen, although Dicer knockout increased apoptosis, most Dicer knockout cells (>96%) survived, whereas viable cells in Dicer floxed group were approximately 98%. Overall, our data suggested that Dicer knockout can lead to concurrent increase in both cell proliferation and apoptosis. Similar findings were reported by Zhang L et al. [25], who found that Dicer ablation induced an increase in prostate cell apoptosis, and, paradoxically, the proliferation of epithelial cells in Dicer knockout mice increased by about three fold, which the authors believe was due to at least in part its compensatory growth. 

Oxidation of fatty acids in the mitochondria is an indispensable physiological process in eukaryotes, including humans. After entering the mitochondria, fatty acids are completely oxidized to water and carbon dioxide through β-oxidation, the TCA cycle, and the ETC [26]. The TCA cycle and ETC also participate in the oxidative metabolism of carbohydrates and proteins [27]. Although the role of Dicer in regulating cellular metabolism of adult C-MSC is largely unknown, Dicer was reported to coordinately regulate lipid metabolism and inflammatory responses in cardiac macrophages by enhancing fatty acid-fueled mitochondrial respiration. Moreover, Dicer gene deletion decreased fatty acid oxidation by modulating β-oxidation, the TCA cycle, and ETC activity in macrophages [28]. We found that Dicer gene deletion in C-MSC resulted in a significant reduction in mitochondrial oxidative metabolism, and increased glycolysis; thus, Dicer appears to be important in maintaining fatty acid oxidative metabolism/β-oxidation in C-MSC.

To investigate the mechanism by which Dicer gene deletion represses mitochondrial oxidative metabolism in C-MSC, we analyzed the expression of β-oxidation, TCA, and ETC-related genes in C-MSC with/without Dicer knockout. We found that Dicer gene deletion significantly reduced the expression of Sirt1, Ppargc1b, Acox3, and Hadhb, without affecting the expression of TCA and ETC-related genes. Ppargc1 is preferentially expressed in tissues with high oxidative capacities, such as heart and brown adipose tissue, and serves as a regulator of mitochondrial function and cellular energy metabolism [29]. Sirt1 deacetylase can increase oxidation rates of fatty acids by the deacetylation and activation of transcriptional coactivator Ppargc1 [30,31]. Acox1, the first enzyme in the β-oxidation pathway, catalyzes the desaturation of acyl-CoAs to 2-trans-enoyl-CoAs [32]. Although Dicer gene deletion in C-MSC did not reduce Acox1 expression, it significantly decreased Acox3 expression in these cells. As a paralog of Acox1, Acox3 contributes significantly to peroxisomal branched-chain fatty acid beta-oxidation [33]. Hadha and Hadhb respectively encode the alpha and beta subunits of the mitochondrial trifunctional protein, which catalyzes the last three steps of mitochondrial beta-oxidation of long-chain fatty acids [34]. Our data indicate that Dicer gene deletion significantly reduces Hadhb, but not Hadha, expression. A recent study demonstrated that Hadhb is directly associated with severe childhood-onset cardiomyopathies [35]. To determine whether similar gene-specific changes would occur in human cells, we knocked down Dicer in human C-MSC (isolated from the right atrial appendage). We achieved approximately 75% reduction in Dicer gene expression in these cells, which resulted in significant downregulation of Sirt1 and Ppargc1, indicating that Dicer also regulates the expression of fatty acid oxidation genes in human C-MSC. On the basis of the concept that Dicer controls the maturation of thousands of small non-coding RNAs, one mRNA can also be targeted by multiple miRNAs. Therefore, the metabolic switch induced by Dicer gene deletion might not be explained by single aberrant miRNA controlling expression of fatty acid oxidation genes in C-MSC, but rather by perturbation of an interaction-regulatory network controlled by thousands of miRNAs [36].

The fetal heart exists in a hypoxic environment and obtains the bulk of its energy via glycolysis. After birth, the “fetal switch” to the oxidative metabolism of glucose and fatty acids has been linked to the loss of the regenerative phenotype [37]. Our study suggests that Dicer-intact C-MSC rely on oxidative metabolism, whereas knocking out Dicer increases glycolysis and inhibits mitochondrial oxidative metabolism, suggesting that Dicer ^−/−^ C-MSC can adapt to a low fatty acid environment. Embryonic stem cells, as well as cancer cells, exhibit high glycolysis rates, and low oxidative phosphorylation to support rapid cell proliferation, irrespective of oxygen tension, a phenomenon known as the Warburg effect [38]. The primary function of aerobic glycolysis is to provide glycolytic intermediates for anabolic reactions in these cells, thereby becoming a metabolic pathway of choice during cell proliferation [39,40]. 

Our study provides evidence that Dicer gene deletion in C-MSC converts cellular metabolism from an oxidative state to a glycolytic state. We anticipate that the decrease in Dicer expression in C-MSC may make C-MSC more adaptable to ischemic myocardium by reducing energy expenditure. Although we observed increased cell proliferation and apoptosis in Dicer knockout C-MSC, we cannot exclude the possibility that transient knockdown of Dicer in donor adult stem cells might improve donor cell survival in ischemic myocardium after cell transplantation. In vivo experiments are needed to test this hypothesis.

## 4. Materials and Methods 

### 4.1. Isolation of Mouse and Human C-MSC 

Dicer floxed mice (The Jackson Laboratory, Bar Harbor, ME, USA) were euthanized, and hearts were harvested and washed with 1× phosphate-buffered saline (PBS). Mouse C-MSC were isolated from these hearts, as previously described [8,9,41], and used at passage 6–10. Human right atrial appendage tissues were collected from patients undergoing cardiothoracic surgery. The collection of discarded right atrial appendages was approved by the Institutional Review Board of Medical College of Georgia at Augusta University. The study was based on the recommendations of the Declaration of Helsinki. Tissues were minced into approximately 1 mm three-sized pieces and digested using 0.1% collagenase IV and 1 U/mL dispase in. Dulbecco’s Modified Eagle Medium F-12 (DMEM/F-12) for 1 h at 37 °C. Then, cardiac explants were collected and incubated on fibronectin/gelatin-coated plates (0.5 mg fibronectin in 100 mL 0.1% gelatin) in DMEM containing 10% fetal bovine serum, 100 U/mL penicillin G, and 100 μg/mL streptomycin. Cells were cultured under these conditions until they became confluent in 7–10 days.

### 4.2. siRNA Transfection

Human C-MSC in 24-well plates were incubated with 20 nM of human Dicer small interfering RNA (siRNA) or scrambled negative control (NC) siRNA (Santa Cruz Biotechnology, Santa Cruz, CA, USA) with Lipofectamine RNAiMax transfection reagent (Thermo Fisher, Waltham, MA, USA) in 1 mL culture medium for 48 h at 37 °C. 

### 4.3. Flow Cytometry

C-MSC were blocked with 5% rat serum and stained respectively with conjugated antibodies, including anti-CD105-APC (BioLegend, San Diego, CA, USA), anti-CD31-APC (BD Biosciences, San Jose, CA, USA), anti-CD45-APC (BD Biosciences), anti-CD140b-PE (BioLegend), or isotype-matched control antibody (BD Biosciences). Flow cytometry analysis of cultured C-MSC was performed with a BD LSRII flow cytometer and BD FACSDiva™ software (8.02, BD Biosciences, San Jose, CA, USA).

### 4.4. Immunofluorescent Staining

For cell staining, C-MSC plated on an 8-well chamber slide (Thermo Fisher Scientific, Waltham, MA, USA) were fixed with 4% paraformaldehyde, followed by the permeabilization with 1% TritonTM X-100. After blocking with 5% goat serum, cells were incubated with rabbit anti-GATA4 (1:100; Aviva System Biology, San Diego, CA, USA) or anti-Ki67 (1:400, Cell Signaling Technology, Danvers, MA, USA) at 4 °C overnight. Secondary antibody incubation with goat anti-rabbit Alexa Fluor 555-conjugated (1:400, Invitrogen, Carlsbad, CA, USA) was performed the following day. To detect cell apoptosis, we performed TUNEL staining using DEAD End TUNEL kit (Promega, Madison, WI, USA) according to the manufacturer’s instructions with modifications [41,42]. Briefly, cells were fixed with 4% paraformaldehyde and permeabilized with 1% Triton X-100. Labeling reactions were performed for 60 min at 37 °C, followed by streptavidin Alexa Fluor 555-conjugated (1:400, Life Technologies, Carlsbad, CA, USA) incubation. Slides were mounted using VECTASHIELD HardSet Mounting Medium with DAPI (Vector Laboratories, Burlingame, CA, USA). Staining was analyzed by a Zeiss 780 laser scanning microscope (Carl Zeiss, Thornwood, NY, USA). The number of Ki67-positive or TUNEL-positive cells were counted in six randomly selected fields.

For mitochondrial staining, C-MSC in 3.5 cm glass-bottom microwell dishes (MatTek, Ashland, MA) were incubated with DMEM containing 100 nM Mitotracker Red CMXRos (Invitrogen, Carlsbad, CA, USA) at 37 °C for 15 min. After fixing with 4% paraformaldehyde, cells were mounted with VECTASHIELD HardSet Mounting Medium with DAPI. Staining was analyzed by a Zeiss 780 laser scanning microscope (Carl Zeiss, Thornwood, NY, USA).

### 4.5. Plasmid Construct and Generation of Recombinant Adenoviruses

The adeno-backbone plasmid (pAdtrack-CMV-CRE) was produced by amplifying 1 kb of CRE cDNA from Puro.Cre plasmid (a gift from Tyler Jacks, Addgene plasmid #17408) via PCR with primers containing Not1 and Xho1 restriction sites on the ends (Forward PCR primer: 5′-AAGGAAAAAAGCGGCCGCGCCACCATGCCCAAGAAGAAGAGGAA-3′. Reverse PCR primer: 5′-CTAGCTCGAGCTAATCGCCATCTTCCAGCA-3′). The CRE fragment was subcloned into the Not1-Xho1 site of the pAdtrack-CMV vector (a gift from Bert Vogelstein, Addgene plasmid #16405). Recombinant adenovirus Adtrack-CMV-CRE and Adtrack-CMV were generated by using the AdEasy-1 expression system, as previously described [43]. Briefly, parental empty pAdtrack-CMV bearing GFP and pAdtrack-CMV-CRE vectors bearing CRE and GFP were first linearized with PmeI and then introduced into BJ5183-AD-1 electroporation competent cells (Agilent Technologies, La Jolla, CA) by electroporation. Recombinant adenoviral plasmids were recovered from *E. coli* and introduced into AD-293 cells by Fugene HD (Roche Applied Science, Indianapolis, IN, USA). The recombinant viruses were propagated in AD-293 cells, purified, and concentrated using the Adeno MINI Purification ViraKit (VIRAPUR, LLC, San Diego, CA, USA). Floxed Dicer C-MSC seeded on dishes were infected with adenoviruses at a multiplicity of infection of 200 for the described experiments.

### 4.6. Isolation and Quantification of Genomic DNA and Messenger RNA

Genomic DNA was extracted from C-MSC with the QIAamp DNA blood mini kit (QIAGEN, Valencia, CA) following the manufacturer’s instructions. 

Total RNA was extracted by RNAzol RT (Molecular Research Center, Inc, Cincinnati, OH, USA) following the manufacturer’s instructions. cDNA was synthesized from total RNA using the RevertAid First Strand cDNA Synthesis kit (Thermo Scientific). The cDNA was used to perform quantitative PCR on a CFX96 Touch Real-Time PCR Detection System (Bio-Rad Laboratories, Hercules, CA, USA) using PowerUp SYBR Green Master Mix (Thermo Fisher, Waltham, MA, USA). Amplification was performed at 50 °C for 2 min, 95 °C for 2 min, followed by 50 cycles of 95 °C for 15 s, and 60 °C for 1 min. The primer sequences are listed in Table 1.

### 4.7. Western Blotting Assay

Western blotting was performed as described previously [8]. Briefly, normalized concentration protein was loaded on a 10% SDS–polyacrylamide gel and separated by electrophoresis. Then, the protein was transferred from the gel to a polyvinylidene difluoride (PVDF) membrane. After blocking with Odyssey blocking buffer (LI-COR Biosciences, Lincoln, NE, USA), the membrane was probed using rabbit anti-Dicer (1:2000, Novus Biologicals, Littleton, CO, USA), rabbit anti-PCNA (1:1000, Cell signaling Technology), rabbit anti-phospho-Histone H3 (1:1000, Cell Signaling Technology), and mouse anti-β Actin (1:5000, Novus Biologicals, Lincoln, NE, USA) at 4 °C overnight. After washing with 1 × TBST, the membrane was incubated for 1 h at room temperature with IRDye 680 goat anti-rabbit IgG or IRDye 800 goat anti-mouse IgG (1:10,000, LI-COR Biosciences). The probed blot was scanned using an Odyssey infrared imager.

### 4.8. Seahorse Analysis of Mitochondrial Respirometry

OCR and ECAR were detected using Seahorse XF 96 Extracellular Flux Analyzer (Agilent Technologies, Lexington, MA, USA) according to the manufacturer’s instructions. Briefly, the cells were plated at 10,000 cells/well in the Seahorse XF Cell Culture Microplate. The sensor cartridge for XF analyzer was hydrated in a 37 °C non-CO_2_ incubator the day prior to experimentation. On the day of the Seahorse assay, the medium was changed to Seahorse XF DMEM base medium, without phenol red, supplemented with 10 mM glucose, 2 mM L-glutamine, and 1 mM pyruvate, pH 7.4, and then metabolic parameters were measured using the Mitochondrial Stress Test Kit (Agilent, Cat. No. 103015-100, Santa Clara, CA, USA). OCR and ECAR were performed using the following protocol after calibration and equilibration: three cycles of wait 3 min, mix 3 min, measure 3 min basal values and then values after each of the following injections (see Figure 3A for a description): 1.25 µM oligomycin (final concentration), 1 µM FCCP (final), and 0.5 µM rotenone/antimycin A (final). Post assay wells were washed, lysed, and a bicinchoninic acid assay (BCA) Protein assay was performed for protein content. Data presented are normalized for total protein per well. Each point represents an average of eight different wells.

### 4.9. Statistics

Results are presented as the mean ± standard error of the mean (SEM). Unpaired Student’s *t*-test was used to compare two groups. A value of *p* < 0.05 was considered statistically significant. Statistical analyses were conducted with GraphPad Prism 7.0 software. 

## 5. Conclusions

Our findings demonstrate an essential function of Dicer to maintain mitochondrial β-oxidation of fatty acids in C-MSC. Dicer gene deletion in C-MSC switched the cellular metabolism from oxidative state to a glycolytic state, likely in part by regulating the expression of essential genes involved in fatty acid beta-oxidation.

## Figures and Tables

**Table 1:** Primer list.

Gene	Sequence (5′–3′)
*β-actin FWD* (mouse)	AGAGCATAGCCCTCGTAGAT
*β-actin REV* (mouse)	GCTGTGCTGTCCCTGTATG
*Dicer FWD* (mouse)	TTACCAGCGCTTAGAATTCCTGGG
*Dicer REV* (mouse)	GTTATTGACAAGGGCAGAGCGCAA
*Sirt1 FWD* (mouse)	GTTGGTGGCAACTCTGATAAATG
*Sirt1 REV* (mouse)	GTCATAGGCTAGGTGGTGAATATG
*Ppargc1b FWD* (mouse)	AGGTGTGAGGGAAGCATAGA
*Ppargc1b REV* (mouse)	CAAAGCCTTCTGGACTGAGTT
*Acox1 FWD* (mouse)	CCTTGGCCAATGCTCTCATTA
*Acox1 REV* (mouse)	CGCAGCAGTATAAACTCTTCCC
*Acox3 FWD* (mouse)	CCCTAGAGAAGCTACGAGAACT
*Acox3 REV* (mouse)	CAGGCAGTTAATCAGCACTAGAA
*Hadha FWD* (mouse)	CCATGTCGGCCTTCTCAAA
*Hadha REV* (mouse)	AGTGAAGAAGAAAGCTCTCACAT
*Hadhb FWD* (mouse)	AGACCATGGGCCACTCT
*Hadhb REV* (mouse)	CTTCTTGGCCAGACTATGAGAAC
*Idh3a FWD* (mouse)	GGCCATCCATCTATGAATCTGT
*Idh3a REV* (mouse)	GTATTCTCCTTCCGTGTTCTCTC
*Ogdh FWD* (mouse)	CATGTATCACCGCAGGATCAA
*Ogdh REV* (mouse)	GGTCTTTCCCATCACGACAG
*Sdhd FWD* (mouse)	GATGCCGACATCGTGGTAAT
*Sdhd REV* (mouse)	GTTACCGACTACGTTCATGGG
*Uqcrq FWD* (mouse)	CTTTGCTGAAATAGCTTGGGAAG
*Uqcrq REV* (mouse)	GAACCTGGCGCGGATAC
*GATA4 FWD* (mouse)	GAGGGTGAGCCTGTATGTAATG
*GATA4 REV* (mouse)	CCTGCTGGCGTCTTAGATTTAT
*NKX2-5 FWD* (mouse)	GCAGTGGAGCTGGACAAA
*NKX2-5 REV* (mouse)	GGTACCGCTGTTGCTTGA
*cTNI FWD* (mouse)	CACCTCAAGCAGGTGAAGAA
*cTNI REV* (mouse)	GCCACTCAGTGCATCGATATT
*MT-ND6 FWD* (mouse)	CACCCAGCTACTACCATCATTC
*MT-ND6 REV* (mouse)	GTTTGGGAGATTGGTTGATGTATG
*POLB FWD* (mouse)	GGCGGATGGTGTACTCATT
*POLB REV* (mouse)	ACTGTGGTGTTCTCTACTTCAC
*β-actin FWD* (human)	CGTAGCACAGCTTCTCCTTAAT
*β-actin REV* (human)	GGACCTGACTGACTACCTCAT
*Dicer FWD* (human)	GGTTCCAGAACTCTGTGCTATAC
*Dicer REV* (human)	AGGCAGTGAAGGCGATAAAG
*Sirt1 FWD* (human)	CACCCACACCTCTTCATGTT
*Sirt1 REV* (human)	CATTACTCTTAGCTGCTTGGTCTA
*Ppargc1b FWD* (human)	GGTAAGGATCGCAGCTTCAC
*Ppargc1b REV* (human)	CCTCTTCCTCCTCCTCTTCTT
*Acox3 FWD* (human)	CCCACAGAGGAGGAGGAAATA
*Acox3 REV* (human)	GCGACTTGGAGAAATGGTCTAA
*Hadhb FWD* (human)	CCAAGAAGGCACAGGATGAA
*Hadhb REV* (human)	GGAAGGACGGATGCCATTAT

## Supplementary Materials

Supplementary materials can be found at https://www.mdpi.com/1422-0067/20/22/5554/s1.

## Abbreviations

BM-MSC	Bone marrow mesenchymal stem cells
Nkx2.5	NK2 homeobox 5
cTNI	Cardiac troponin-I
MT-ND6	mitochondrially encoded NADH: ubiquinone oxidoreductase core subunit 6
POLB	DNA polymerase beta
TUNEL	Terminal deoxynucleotidyl transferase (TdT) dUTP nick-end labeling
TCA	Tricarboxylic acid
ETC	Electron transport chain
C-MSC	Cardiac mesenchymal stem cells
miRNAs	microRNAs
OCR	Oxygen consumption rate
ECAR	Extracellular acidification rate
Sirt1	Sirtuin 1
Ppargc1b	Peroxisome proliferator-activated receptor gamma coactivator 1 beta
Acox1	Acyl-CoA oxidase 1
Acox3	Acyl-CoA oxidase 3
Hadha	Hydroxyacyl-CoA dehydrogenase trifunctional multienzyme complex subunit alpha
Hadhb	Hydroxyacyl-CoA dehydrogenase trifunctional multienzyme complex subunit beta
Idh3a	Isocitrate dehydrogenase (NAD(+)) 3 alpha
Ogdh	Oxoglutarate dehydrogenase
Sdhd	Succinate dehydrogenase complex subunit D
Uqcrq	Ubiquinol-cytochrome C reductase complex III subunit VII
