# Supplementary material for: RNAase III-Type Enzyme Dicer Regulates Mitochondrial Fatty Acid Oxidative Metabolism in Cardiac Mesenchymal Stem Cells
Source: Int J Mol Sci. 2019 Nov 7;20(22):5554. doi: 10.3390/ijms20225554 (PMC6888515; doi:10.3390/ijms20225554)
Supplement: Supplementary file 1 [file ijms-20-05554-s001.pdf]

Supplemental Figure 1

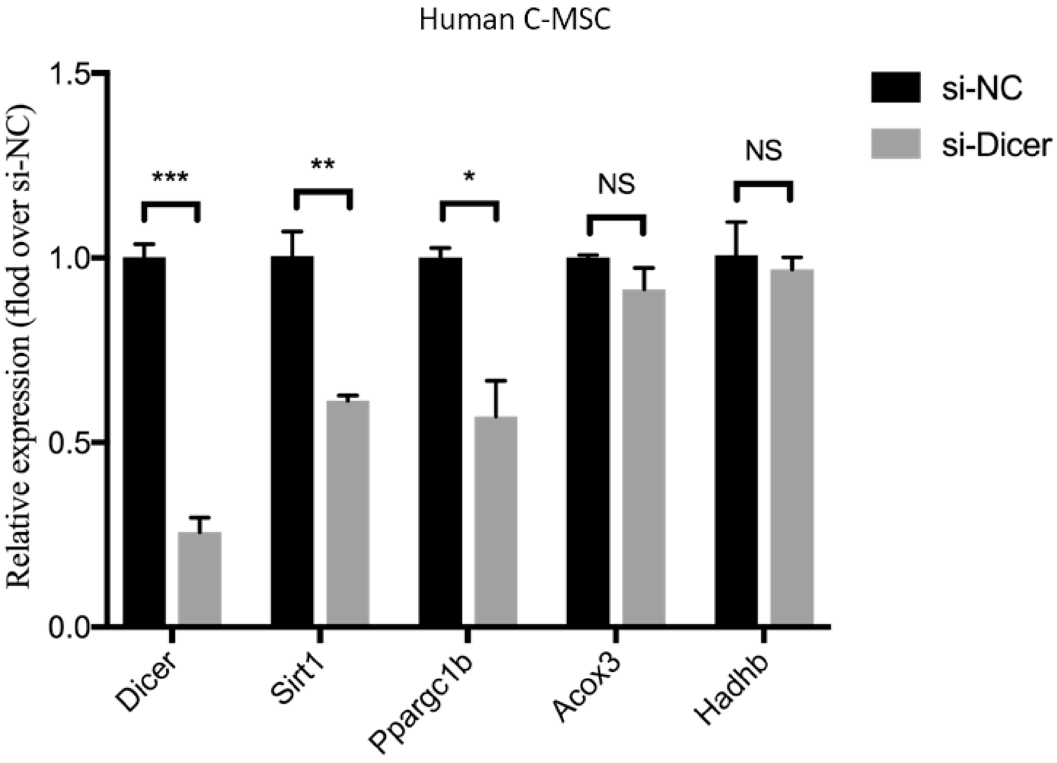

Supplemental Fig. 1. Comparison of relative mRNA levels of genes related to mitochondrial fatty acid oxidation in Dicer siRNA (si-Dicer) and negative control siRNA (si-NC) treated human C-MSC. The amount of mRNA was normalized using β-actin. Results are shown as mean ± SEM (n=3), NS $p > 0.05$, * $p < 0.05$, ** $p < 0.01$, *** $p < 0.001$.
